# A randomized controlled trial to assess if changing sleep timing can improve glucose metabolism in people with prediabetes and type 2 diabetes

**DOI:** 10.1186/s13063-024-08329-w

**Published:** 2024-07-12

**Authors:** Emma J. Bouman, Romy Slebe, Dirk Jan Stenvers, Petra J. M. Elders, Joline W. J. Beulens, Femke Rutters

**Affiliations:** 1 Amsterdam UMC location Vrije Universiteit Amsterdam, Epidemiology and Data Science, Meibergdreef 9, Amsterdam, The Netherlands; 2 Amsterdam Public Health, Health Behaviors & Chronic Diseases, Amsterdam, The Netherlands; 3 Amsterdam UMC location University of Amsterdam, Endocrinology and Metabolism, Meibergdreef 9, Amsterdam, The Netherlands; 4 Amsterdam Gastoenterology, Endocrinology Metabolism, Amsterdam, The Netherlands; 5 Amsterdam UMC location Vrije Universiteit Amsterdam, Endocrinology and Metabolism, Meibergdreef 9, Amsterdam, The Netherlands; 6 Amsterdam UMC location Vrije Universiteit Amsterdam, General Practice, Meibergdreef 9, Amsterdam, The Netherlands

**Keywords:** Social jetlag, Circadian rhythm, Type 2 diabetes mellitus, Glycemic control, Metabolic control, Randomized controlled trial

## Abstract

**Background:**

Social jetlag is a chronic disruption of sleep timing that is characterized by different sleep timing during workdays and free days. Social jetlag has been associated with disturbed glucose metabolism, insulin resistance, and increased risk of metabolic syndrome and type 2 diabetes. In this study, we aim to investigate whether a combination of bright light therapy in the morning, bright light reduction in the evening and sleep advance instructions for 3 weeks reduces social jetlag and if this results in improvement of glycemic and metabolic control, sleep, mood and quality of life after 3 and 12 weeks in people with prediabetes and type 2 diabetes and to assess possible mediators, compared to regular sleep habits.

**Methods:**

In this randomized controlled trial, 60 people with prediabetes or type 2 diabetes with > 1 h social jetlag will be recruited. The intervention consists of bright light therapy (5000 lx) emitted by Vitamine-L (Lumie, UK) for 30 min each morning, combined with the advice to follow sleep advance instructions and to wear bright light-dimming goggles every evening for a period of 3 weeks. The control group adheres to their regular sleep habits and conditions. The primary outcome is glycated hemoglobin (HbA1c) after 12 weeks comparing the intervention and control in an intention-to-treat analysis. Secondary outcomes at 3 and 12 weeks are (1) social jetlag; (2) insulin sensitivity, fasting blood glucose, glucose-lowering medication use, and frequency of perceived hypoglycemia; (3) metabolic outcomes, including body mass index (BMI), waist circumference, body fat percentage, and blood pressure; (4) mood, including depression, fatigue and anxiety (measured with questionnaires); and (5) quality of life measured using EQ5D questionnaire. To assess other factors that might play a role as possible mediators, we will measure (para)sympathetic nervous system activity assessed with ECGs and electrochemical skin conductance tests, sleep quality and sleep phase distribution assessed with a sleep measuring headband (ZMax), the Dim Light Melatonin Onset in saliva samples (in a subgroup) at 3 and 12 weeks, the feeling of satiety and satiation with a 10-cm visual analog scale (VAS), diet using a food frequency questionnaire, and physical activity using an accelerometer (ActiGraph).

**Discussion:**

Social jetlag can contribute to poorer glycemic control and metabolic control in those with type 2 diabetes. With this intervention, we aim to reduce social jetlag and thereby improve glycemic and metabolic control. This could offer a way to improve overall population health and to reduce the disease burden of type 2 diabetes.

**Trial registration:**

ISRCTN registry ISRCTN11967109. Registered on 9 May 2024.

## Introduction

### Background and rationale {6a}

Social jetlag is a chronic disruption of sleep timing. It is caused by a discrepancy between biological rhythms and social engagements and is characterized by different sleep timing during workdays and free days [1]. Unlike sleep disruption caused by performing shift work or by traveling through different time zones, social jetlag is subtle but highly prevalent with estimates of over 70% in students and over 50% in the working population [2, 3]. Social jetlag usually starts during adolescence and remains present throughout the working career [1–4].

Social jetlag is associated with several pathologies such as mental health, cardiovascular disease, and type 2 diabetes [2]. In our recent meta-analysis, with data from 68 studies, we found that social jetlag is significantly associated with higher HbA1c levels, higher odds of obesity, and increased BMI [5]. In our cross-sectional study in people with type 2 diabetes, we showed that in working people, a high social jetlag was associated with a 1.87 mmol/mol (95% CI: 0.75 to 2.99) higher HbA1c and 5.81 mmHg (95% CI: 4.04 to 7.59) [6] higher blood pressure.

In other words, social jetlag can contribute to poorer glycemic and metabolic control in those with type 2 diabetes. As type 2 diabetes is a highly prevalent disease that causes a high burden of disease with over 400 million people affected worldwide [7], reducing social jetlag could offer a way to reduce this disease burden and improve overall population health.

Previous research has shown that it is possible to reduce social jetlag. For example, in the study by Geerdink et al. (2016) [8], 42 participants with > 1 h social jetlag were either exposed to a high-intensity blue light (intervention) or amber light exposure (placebo) in the morning for 9 days, combined with a sleep advancing scheme. This intervention advanced bed times by at least 45 min and wake-up times by at least 30 min, thereby changing the timing of sleep but not the duration, resulting in a reduction of social jetlag. Moreover, blue light was more effective in advancing the circadian clock, assessed through Dim Light Melatonin Onset (DLMO), compared to amber light in the placebo group and still had a significant effect 1 week after the intervention was stopped, with participants from the blue light group still waking up 30 min earlier. Overall, this study showed that blue light combined with a sleep advancing scheme for only 1 week is effective in reducing social jetlag [8].

The only other study assessing methods to reduce social jetlag was the study by Zerbini et al. (2018) [9]. For 2 weeks, they exposed 76 participants with at least 1.5 h social jetlag to either control (no intervention) or to blue light reduction in the evening condition. They showed a 36-min advance in sleep onset on workdays, but not on free days, an advanced DLMO, and a trend towards longer sleep in the intervention group compared to the control group, although social jetlag was not reduced [9].

Although it is possible to reduce social jetlag, currently there are no studies on the metabolic effects of these types of social jetlag reduction interventions. Therefore, our aim is to investigate whether a reduction in social jetlag for 3 weeks can improve glycemic and metabolic control, sleep, mood, and quality of life in people with prediabetes and type 2 diabetes.

### Objectives {7}

The primary objective of this study is to investigate whether a combination of bright light therapy in the morning, bright light reduction in the evening and sleep advance instructions for 3 weeks to reduce social jetlag results in an improvement of glycemic control in people with prediabetes and type 2 diabetes, compared to regular sleep habits. As a secondary objective, we assess the effect of the intervention on additional glycemic outcomes, metabolic control, sleep, mood, and quality of life.

### Trial design {8}

This study is a randomized controlled intervention study with two conditions. Stratified randomization allocates participants 1:1 to control or intervention conditions with equal distribution of sex, age, and social jetlag.

## Methods: participants, interventions, and outcomes

### Study setting {9}

Sixty participants with prediabetes and type 2 diabetes and > 1 h of social jetlag [10, 11] are recruited from our cohorts including the Hoorn Study [12] and the Hoorn Diabetes Care System Cohort [13]. We will recruit participants from these studies who previously consented to being (re)-approached for additional research. Participants will be invited to our clinical trial unit at the research center in Hoorn, which is part of the Amsterdam UMC.

### Eligibility criteria {10}

#### Inclusion criteria

- Social jetlag (> 1 h) (calculated as the difference between midpoint of sleep during work days and free days)
- Prediabetes or type 2 diabetes: HbA1c > 39 mmol/mol (5.7%), fasting plasma glucose > 5.6 mmol/l, or 2-h OGTT > 7.8 mmol/l (according to the American Diabetes Association) or the use of any glucose-lowering medication
- Informed consent (at inclusion for cohort study) to be contacted for future research
- Willing to comply with the study procedures

#### Exclusion criteria

- Having any other form of diabetes not considered type 2 diabetes (e.g., steroid-induced diabetes, diabetes type 3c, MODY, LADA).
- Excessive alcohol use (> 14 alcoholic consumptions per week)
- Having crossed more than 1 time zone in the month prior to participation
- Working evening or shifts more than once per month
- Unable to provide written informed consent
- Visually impaired (partial or total inability of visual perception)

### Who will take informed consent? {26a}

Written informed consent will be obtained and signed by the coordinating researcher or by a researcher/research nurse on their behalf before the start of any study-related assessments.

### Additional consent provisions for collection and use of participant data and biological specimens {26b}

Not applicable.

## Interventions

### Explanation for choice of comparators {6b}

For the control condition, participants will receive advice on healthy sleep habits and remain the same sleeping conditions and habits. Due to the nature and design of the study, blinding of the researchers or participants is not possible.

### Intervention description {11a}

The intervention condition is a combination of evidence-based techniques to realign social jetlag. It consists of three components: bright light therapy in the morning, sleep advance instructions, and bright light reduction in the evening.

First, for the bright light therapy, a Vitamin-L lamp (Lumie, UK) will be placed at 30 cm before the participant, yielding 5000 lx (https://www.lumie.com/products/vitamin-l), which is similar to outside light levels on a clouded day. The participant does not have to look straight into the light. Participants are requested to expose themselves to 30 min of bright light each morning by sitting in front of the lamp, while working on their computer or reading. Participants will receive a Vitamin-L lamp together with instructions for use. The Vitamin-L lamp is certified to ISO 13485 Medical Devices, QMS standard. Lights must pass rigorous safety tests and be supported by clinical research to meet the Medical Devices Directive EC93/42/EEC. They are approved for use for light therapy at home. Thus, in the present protocol, the lights will be used for the purpose they are designed for (i.e., administration of light). Due to recent insights about possible interactions of different photoreceptors in the non-visual effects of light [14], we believe that it is probably most efficient to use full spectrum bright light in the morning, rather than select a certain specific wavelength, such as blue light only.

Second, study participants are told to sleep more regularly, with similar sleep onset and sleep offset times on weekdays and weekend days. To support them with this, participants will follow a personal sleep-advancing scheme [8]. Based on self-report of the participant, the habitual sleep onset and habitual sleep offset will be calculated as the average of all days (week and weekend days). Then participants are asked to give their preferred sleep offset for all days of the week (similar for week and weekend days); preferred sleep onset is calculated as sleep offset − preferred sleep duration between 7 and 8 h. To advance sleep time with an average 20 min, the first 3 days, the light therapy will be scheduled 10 h after preferred sleep onset. On days 4–6, the light therapy is scheduled 9 h after preferred sleep onset and days 7–9 the light therapy 8 h after preferred sleep onset, if the earlier sleep onset is 3 h earlier. After the preferred sleep offset and onset are reached, the new schedule will be continued during the rest of the 3 weeks intervention.

As final part of the intervention, participants receive blue-light dimming goggles with instructions to wear them every evening from 18.00 h until they go to sleep [15]. When wearing the goggles they can still perform their usual activities, such as watching television or reading, with screens of electronic devices in dimmed modus [15, 16] as well as room lighting (dim lighting of the room, curtains closed).

### Criteria for discontinuing or modifying allocated interventions {11b}

Not applicable.

### Strategies to improve adherence to interventions {11c}

To improve adherence to the protocol and assess compliance, participants will send in a sleep diary of 7 days and wear an accelerometer (ActiGraph) as well as a light logger (Pendant Light 64 K Data Logger, HOBO CE) for 7 days after 1 week of the intervention program. The light logger is worn as pendant (2 by 3 cm) on a necklace outside the clothes, only taken off in water-based activities. The accelerometer and sleep diary will be used to assess adherence to the sleep schedule and the use of the light-dimming goggles, and the light logger will be used to assess compliance to the use of the bright light therapy. The control group receives no instructions and will continue with their usual sleep habits and conditions.

### Relevant concomitant care permitted or prohibited during the trial {11d}

There are no restrictions regarding concomitant care and medication use during this study.

### Provisions for post-trial care {30}

After the trial has finished, participants from the intervention group can keep adhering to their sleep instructions and can keep the blue light-dimming goggles. The Vitamin L lamp has to be returned. Also, participants from the control group can request a personal sleep schedule and blue light dimming goggles after the trial is finalized.

### Outcomes {12}

#### Primary outcomes

The primary outcome is HbA1c (mmol/mol) determined with turbidimetric inhibition immunoassay (Cobas c501, Roche Diagnostics, Mannheim, Germany) after 12 weeks.

#### Secondary outcomes

As secondary outcome measures, we will determine social jetlag measured using an adapted version of the Munich ChronoType Questionnaire (MCTQ) [10, 17] and sleep times from accelerometer data (ActiGraph) and EEG data from the sleep headband (ZMax) after 3 and 12 weeks. We will also determine other measures of glycemic control after 3 and 12 weeks. We will measure HOMA-IR determined from fasting insulin levels using an anti-body kit (AutoDELFIA Insulin kit; Wallac Oy), fasting blood glucose determined in fluorinated plasma with the UV test using hexokinase (Cobas c501, Roche Diagnostics); self-reported perceived hypoglycemia determined using a self-constructed questionnaire; and diabetes medication use assessed from the participants medication overview and described as type (ATC code), timing and dosage). Furthermore, we will measure metabolic parameters including anthropometrics (body weight, body fat percentage, and waist circumference); blood pressure (described as a continuous variable or dichotomous as hypertension, defined as systolic blood pressure and diastolic blood pressure ≥ 140/90 and/or use of hypertensive medication) Finally, mood, depression, fatigue, anxiety, and quality of life will be assessed using PROMIS questionnaires.

#### Possible confounders and mediators

Participants will self-report age, sex, education status, stress level, work status and medication use, diet, alcohol consumption, smoking, feeling of satiety, and satiation. Autonomous nervous system activity will be assessed using an electrochemical skin conductance test, and cardiovascular health will be assessed using a standard resting 12-lead ECG and coded according to the Minnesota coding system. Sleep rhythms, quality, and sleep phases will be assessed using at-home EEG measurements using the ZMax headband [18], accelerometers (ActiGraph), sleep diaries, and the MCTQ questionnaire. Additionally, Dim Light Melatonin Onset was assessed using 6-point saliva measurements and 3-point saliva measurements for cortisol awakening reaction (in a subgroup) and physical activity (total activity levels, moderate to vigorous physical activity, and total sedentary time) measured using an accelerometer.

### Participant timeline {13}

**Fig. 1:**
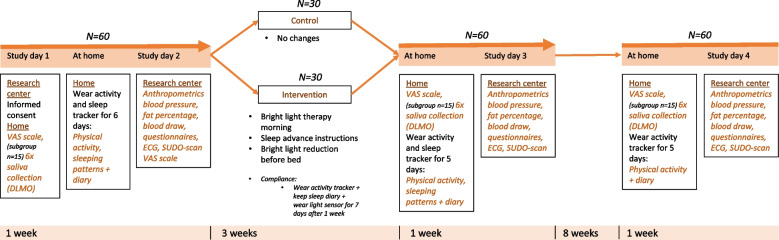
Timeline of study visits, intervention, and measurements of the social jetlag trial

**Fig. 2:**
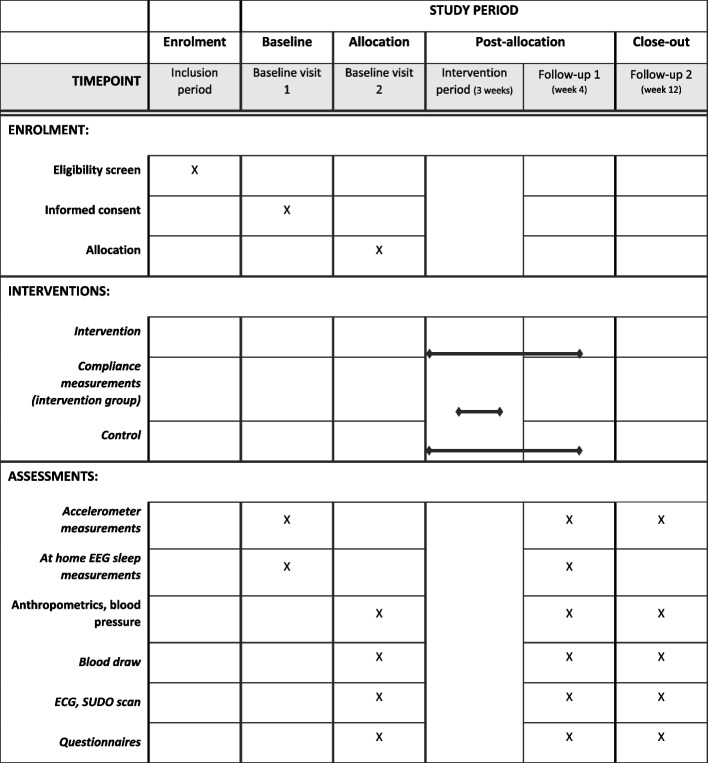
Schedule of enrolment, interventions, and assessments social jetlag trial

Figures 1 and 2 present a timeline of the enrollment, study visits, intervention period, and measurements taken. Participants are invited to the research center in the afternoon and informed consent is signed. Then participants will go home and will fill out VAS scales about satiety and satiation before and after having dinner. A subgroup (*n* = 15) will be asked to wear light-dimming goggles for one evening and collect six saliva samples for melatonin onset assessment. After that, all participants will wear an activity tracker (ActiGraph) at home during regular activities and a sleep monitoring headband (ZMax) at night for a period of 6 days and they will fill in a sleep diary during this period. During the second study day at baseline, anthropometrics and blood pressure will be measured and blood will be drawn to determine HbA1c levels as well as fasting insulin and glucose levels. Participants will also fill in a VAS scale on satiety and satiation and several questionnaires. After that, an ECG and electrochemical skin conductance test will be performed. At the end of the visit and after the participants receive breakfast, they will be randomly assigned to either the control or intervention group and they will receive information on the intervention program or control condition.

To improve adherence to the protocol and assess compliance, 1 week after starting the intervention, participants will keep a sleep diary of 7 days and wear an accelerometer as well as a light tracker.

After a period of 3 weeks, all participants will wear an activity tracker and a sleep-monitoring headband for 5 days and fill in a sleep diary. After that, participants will be invited back to the research center once for the same measurements as the second day of the baseline visit (anthropometrics, blood pressure, blood draw, ECG and SUDO, and questionnaires). Twelve weeks after the start of the intervention, they will wear an activity tracker for 5 days again and they will come back to the research center for a final visit repeating the same measurements.

### Sample size {14}

We calculated the sample size based on the primary outcome, namely HbA1c after 12 weeks. To date, no previous study investigating these social jetlag-reducing interventions has assessed metabolic outcomes. Previous lifestyle interventions in the target population show a change in HbA1c over a period of 3–6 months of 0.2–0.75% [19, 20]. We hypothesize a difference of − 0.25% in HbA1c levels between the two groups after the current intervention. To assess a change of − 0.25% ± 0.25 in HbA1c level, with an *α* of 0.05 and *β* of 0.1 the (corrected) number of participants needed is 23 persons per group, with an anticipated 25% drop-out, this results in 30 participants per group [19, 20].

### Recruitment {15}

Participants from our cohorts, who gave consent for future research and who are eligible for this study will be invited. Eligible people will receive detailed written information. They will receive an invitation letter together with the participant information. After 2 weeks, participants will be contacted by telephone by the researcher to discuss participation and plan the study visit. Before the start of the first measurements, the researcher will discuss the study procedures: the purpose, nature, and duration of the research and the related risks and benefits, as well as the consent. The researcher will answer all questions and obtain a written informed consent before the start of the study. This informed consent form will have to be signed at the research center in the presence of one of the investigators or research nurses. Before signing of the informed consent, no measurements will be performed and no invasive questions will be asked.

### Assignment of interventions: allocation

#### Sequence generation {16a}

Randomization will be performed automatically by the Electronic Data Capturing software (Castor EDC) [21]. Blocked randomization will be used to ensure a good balance of participant characteristics in each group. As sex and age have a strong influence on sleep behavior and also on metabolic and glycemic parameters, randomization will be stratified on sex, two strata of age, and two strata of social jetlag (based on levels in our source population) to prevent imbalance. Allocation will be determined by using a computerized random number generation process.

#### Concealment mechanism {16b}

Not applicable.

#### Implementation {16c}

After the baseline measurements are finished on study day 2, participants will be notified whether they are randomized to the intervention or control condition. Participants in the control condition will receive advice on sleep hygiene but are not instructed to change their sleeping conditions and habits. The participants in the intervention group will receive instructions and all materials needed to follow the intervention protocol. They will also receive materials and instructions for the compliance measurements that they will conduct independently from home.

### Assignment of interventions: blinding

#### Who will be blinded? {17a}

Blinding of the research team and participants to treatment allocation is not possible due to the nature of the intervention. Statistical analyses will be performed by a blinded independent researcher.

#### Procedure for unblinding if needed {17b}

Not applicable because the research team and participants are not blinded.

## Data collection and management

### Plans for assessment and collection of outcomes {18a}

HbA1c will be determined from a fasting blood sample after 12 weeks as a measure for long-term glucose control. All data collected during the study visits will be entered into the eCRF (Castor EDC) directly afterwards. Lab results will be entered in the eCRF as soon as they are determined by the laboratory. Finally, the questionnaires will partly be filled in by the participants using Castor EDC and will partly be on paper and subsequently entered in Castor EDC by the research team.

### Plans to promote participant retention and complete follow-up {18b}

As we will recruit participants from our existing study cohorts, they will already be familiar with participating in scientific research. To promote participant retention and follow-up, participants receive written appointment confirmations at home.

### Data management {19}

We will use Castor EDC as a clinical data management system to capture all collected data in electronic case report forms (eCRF) [21]. Data validation checks and notifications are embedded in the eCRF to ensure correct data entry. All original study documents will be filed in the Trial Master File (TMF) and Investigator Site File (ISF).

### Confidentiality {27}

The handling of personal data complies with the Dutch Personal Data Protection Act (AVG/GDPR). The data of all participants that will participate in the present study will be coded. All participants have an identification number, consisting of four numbers, obtained during previous studies. These numbers cannot be traced to the person without the key, which is stored separately. The key document is only available to the participating investigators. Participants can request the information stored in their CRF.

### Plans for collection, laboratory evaluation, and storage of biological specimens for genetic or molecular analysis in this trial/future use {33}

All biological samples collected during this study will be analyzed during or at the end of the study. All remaining materials will be destroyed afterwards.

## Statistical methods

### Statistical methods for primary and secondary outcomes {20a}f

Baseline characteristics will be described by the intervention arm as mean ± standard deviation (SD), as the median and interquartile range (if not normally distributed), or as numbers and percentages if categorical. Statistical analysis will be performed using RStudio version 4.3.0. We will conduct intention-to-treat analysis for primary and secondary outcomes using linear regression analysis, adjusting for baseline values. We will also adjust for the use of diabetes medication and perform sensitivity analyses on changes of medication use during the follow-up period of the study. Additionally, we will assess differences in intervention compliance and effect according to seasonality. Not normally distributed outcomes will be log transformed. We will assess effect modification by working status and chronotype. We will consider the chance of false positive findings when interpreting the results from testing our multiple secondary outcomes. If there is (despite randomization) an imbalance in participant characteristics, a sensitivity analysis will be conducted to assess the effect of the imbalance by adjusting for these variables.

### Interim analyses {21b}

Interim analyses will not be performed for this study.

### Methods for additional analyses (subgroup) {20b}

To determine the magnitude of the possible mediating processes (DLMO, satiety, physical activity, and/or energy intake) between the intervention and the outcomes, parallel multiple mediation analysis is used.

### Methods in analysis to handle protocol non-adherence and any statistical methods to handle missing data {20c}

Missing data will be investigated for all variables and multiple imputation by predictive mean matching will be used to address missing data if the prevalence exceeds 5%. Ten imputation sets with 50 iterations will be performed and results will be gained from pooled estimates.

To assess the influence of non-adherence to the intervention, we will conduct per-protocol analyses (participants with at least 80% adherence according to the sleep diary, accelerometer, and light tracker scores).

### Plans to give access to the full protocol, participant-level data and statistical code {31c}

After finalization and publication of the study, access to the protocol and statistical code can be requested.

## Oversight and monitoring

### Composition of the coordinating center and trial steering committee {5d}

Dr. F. Rutters is the primary investigator (PI) of this study. Coordinating researchers E. Bouman and R. Slebe will be performing the study visits at the research center together with research nurses.

### Composition of the data monitoring committee {21a}

There is no data safety monitoring board (DSMB) or safety committee established for this study given its low-risk profile. On-site monitoring of the study site will be performed regularly by an independent monitor of the Clinical Monitoring Center (CMC) of the Amsterdam UMC.

### Adverse event reporting and harms {22}

Given the noninvasive nature of the study procedures and intervention, we do not expect any serious adverse reactions. Furthermore, events reported spontaneously by participants or observed by the investigator that have a prolonged effect on the physical wellbeing of the participant (e.g., sustained injury caused by a conducted study measurement or sustained injury incurred during the study visit but unrelated to the study measurements) will be reported. Adverse events will be reported to the local ethical committee in the annual progress report.

### Frequency and plans for auditing trial conduct {23}

The study site will be monitored by an independent monitor of the CMC of the Amsterdam UMC. They will review the study files, the original source documents, and the eCRFs to verify correct data collection and processing.

### Plans for communicating important protocol amendments to relevant parties {25}

Substantial changes to the protocol will be submitted for review to the ethical committee in an amendment. If applicable, the participant information will be adjusted accordingly. Additionally, study forms, operating manuals, and the trial registration will be updated. The current version of the study protocol (version 3.0, 21 February 2024) is approved on 14 March 2024.

### Dissemination plans {31a}

We aim to publish the study results in an international, peer-reviewed medical journal.

## Discussion

This paper describes the background and study design of a randomized controlled trial aimed at reducing social jetlag and improving metabolic and glycemic control in people with prediabetes and type 2 diabetes. We hypothesize that our 3-week intervention program will be able to significantly reduce participant’s social jetlag compared to the control condition. Consequently, we expect to find improvement in insulin sensitivity after 3 weeks and a reduction in HbA1c after 12 weeks in people with prediabetes and type 2 diabetes.

This is the first intervention study specifically aimed at reducing the metabolic and glycemic burden caused by social jetlag. To date, several studies have been able to reduce social jetlag [8, 9]. High-intensity light exposure compared to amber light in the morning for several days, combined with a sleep-advancing scheme was able to reduce social jetlag and shift the circadian clock [8]. In addition, blue light reduction in the evening significantly advanced sleep timing on work days [9].

Given these previous results, we expect that we are able to reduce social jetlag in our study population with our intervention protocol that combines several aspects of previously used interventions. In addition, by improving social jetlag we expect to improve glycemic and metabolic control, through improved insulin sensitivity and reduced HbA1c levels in people with prediabetes and type 2 diabetes. If we succeed, this could contribute to reducing the overall burden of metabolic disease and diabetes. Other interventions aimed at improving circadian rhythm through exercise and diet have already been able to improve weight, blood pressure, glycemic control, and lipid levels in people with type 2 diabetes and metabolic syndrome [22–25].

Especially people with prediabetes or early-stage type 2 diabetes could benefit from this intervention to reduce their social jetlag. As these people are at high risk of developing or deteriorating in their diabetes, reducing the metabolic and glycemic burden could possibly help them reverse this disease progress and return to a healthy metabolic state. Additionally, given its exceptionally high prevalence of 50–70% in the general population, social jetlag is an important public health concern to tackle [2, 3]. Educating people at risk about the importance of regular sleep timing and the possibilities to reduce social jetlag to lower glycemic and metabolic burden could increase the implications of our study outcomes.

Strengths of our study design are the elaborate measurements of sleep timing and also repeated measurements of sleep quality using an EEG sensor headband that will provide in-depth insight into participants’ sleep quality. Additionally, we will follow up with participants 8 weeks after the intervention period for a final assessment of long-term glycemic control (HbA1c). Limitations of this study are the inability to use a double-blind study design due to the nature of the intervention. As the intervention is behavioral, the control group might also change their behavior somewhat, such as their sleep rhythms, physical activity, or dietary patterns. This may lead to a larger effect. However, this effect can also be expected in the intervention group and might be an additional working mechanism.

If this study shows that social jetlag can be reduced and metabolic and glycemic control can thereby be improved in people with prediabetes and type 2 diabetes, this may offer a new possibility for lifestyle interventions in this group.

## Trial status

The current trial status is ongoing. The study started on 15 April 2024 (study protocol version 3.0, 21 February 2024). Recruitment is expected to be completed in 2025.

## Data Availability

The data, code and other materials that underlie the results reported in this article are available from hoornstudy@amsterdamumc.nl upon reasonable request to the Hoorn Steering Committee.

## Abbreviations

ATC code: Anatomical Therapeutic Chemical code
BMI: Body mass index
CMC: Clinical Monitoring Center
DLMO: Dim Light Melatonin Onset
DSMB: Data Safety Monitoring Board
ECG: Electrocardiogram
eCRF: Electronic Case Record Form
EDC: Electronic Data Capture
EEG: Electroencephalogram
GDPR: General Data Protection Regulation; in Dutch: Algemene Verordening Gegevensbescherming (AVG)
HbA1c: Glycated hemoglobin
HOMA-IR: Homeostatic Model Assessment for Insulin Resistance
ISO: International Organization for Standardization
MQTC: Munich Chronotype Questionnaire
MREC: Medical research ethics committee; in Dutch: medisch-ethische toetsingscommissie (METC)
OGTT: Oral glucose tolerance test
PI: Principal Investigator
PROMIS: Patient-Reported Outcomes Measurement Information System
QMS: Quality management system
SD: Standard deviation
TMF: Trial Master File
VAS: Visual analog scale
